# The effect of elastic-band resistance training on fecal microbiota and derived metabolites of aged individuals with possible sarcopenia

**DOI:** 10.3389/fmed.2026.1762454

**Published:** 2026-03-26

**Authors:** Yushuang Duan, Liduan Wang, Hongxue Cui, Zilong Fang, Yifan Lu, Zhongguang Sun

**Affiliations:** 1 Shandong Second Medical University, Weifang, China; 2 Affiliated Hospital of Shandong Second Medical University, Weifang, China; 3 Beijing Sport University, Beijing, China

**Keywords:** elastic-band resistance training, gut microbiota, physical function, possible sarcopenia, SCFAs

## Abstract

**Background:**

Individuals with possible sarcopenia exhibit altered microbiota profiles and poor intestinal metabolism. Exercise training is linked to changes in gut microbiota and has been proposed to enhance the quality of aging skeletal muscle.

**Aims:**

In older adults with possible sarcopenia, the study aimed to determine if elastic-band resistance training modulates gut microbiota and its generated metabolites and investigate the underlying relationships with physical function.

**Methods:**

Thirty-one volunteers with possible sarcopenia were randomly assigned to either the control group (CG, *n* = 17) or the intervention group (RG, *n* = 14), which underwent 24 weeks of elastic-band resistance training. Physical function, body composition, and blood and fecal samples were collected from each patient at baseline and 24 weeks. Enzyme-linked immunosorbent assay (ELISA) was used to evaluate protein metabolism regulatory factors, targeted metabolomics was used to quantify short-chain fatty acid (SCFA) levels, and metagenomic sequencing was used to analyze the composition of the fecal microbiota.

**Results:**

The gait speed (GS), arm curl test (ACT), 2-min step test (2MST), and timed up-and-go test (TUGT) all showed notable improvements in the RG. The RG also showed lower serum levels of tumor necrosis factor-*α* (TNF-α) and higher plasma concentrations of acetate and propionate. Following the intervention, the RG displayed decreased abundances of *Eisenbergiella* and *Eggerthella* and increased abundances of the genus *Bacillus*. *Eggerthella* abundance was inversely connected with 2MST performance, whereas the change in propionate level was positively correlated with 2MST, TUGT, GS, and appendicular skeletal muscle index (ASMI).

**Conclusion:**

The elastic-band resistance training effectively improved physical function, modulates gut microbiota and SCFAs. The results revealed the physiological mechanisms by which gut microbiota and SCFAs regulate aging muscle health, providing scientific support for possible sarcopenia prevention and treatment via gut-muscle axis bidirectional crosstalk.

**Clinical trial registration:**

https://www.chictr.org.cn/index.html.

## Introduction

Nearly three decades ago, Rosenberg first coined the term “sarcopenia” to characterize age-dependent reduction in skeletal muscle (1). As a geriatric syndrome marked by the gradual loss of muscle mass, strength, and physical function, sarcopenia plays a pivotal role in driving declines in physical performance among older adults (2). The important role that muscle quality plays in age-related functional loss is shown by the fact that skeletal muscular strength falls more quickly than muscle mass (3). Additionally, skeletal muscular strength often gains more from resistance training than lean body mass, suggesting that the significant decline in age-associated strength cannot be entirely explained by muscle mass alone (4). Skeletal muscle strength rather than skeletal muscle mass has been linked to mortality risk in epidemiological studies (5), and age-related reductions in skeletal muscle strength and physical performance are significant drivers of poor health outcomes in older populations (6). The term “possible sarcopenia,” which is defined as decreased muscle strength with or without reduced physical performance, was first used in 2019 by the Asian Working Group for Sarcopenia (AWGS) (7). In order to assist proactive health management and enable early identification of those at risk, this category was specifically created. The clinical relevance of these age-related physiological changes has been highlighted by large-scale longitudinal investigations that have proven that decreased muscle mass or handgrip strength (HGS) predicts mortality and the emergence of chronic illnesses (1).

There is growing evidence that the gut microbiota and skeletal muscle interact in both directions, forming a regulatory system known as the “gut-muscle axis” (8). By regulating intestinal barrier dysfunction, metabolic balance, endocrine signaling, chronic low-grade inflammation, and immunological competence—all of which are essential to muscle physiology—the gut microbiota has a significant impact on the musculoskeletal system (9). Age-related muscular weakness and sarcopenia have been linked to dysregulated microbial composition (10). Our previous study showed that older adults who have possible sarcopenia have markedly diminished physical function, along with clear changes in the abundance of bacterial genera that produce short-chain fatty acids (SCFA), such as *Faecalibacterium*, *Eubacterium*, *Intestinimonas*, and *Roseburia*. Changes in physical function measurements are closely correlated with these microbial modifications (11), indicating that gut microbiota regulation may be a feasible treatment approach for musculoskeletal illnesses.

Many health advantages of exercise are well known, and new research indicates that these benefits may be partially mediated by exercise’s ability to alter the gut microbiota (12). Frequent exercise helps maintain a balanced microbiome composition, especially by enriching taxa that support healthy muscles. Regular exercise may preserve a homeostatic balance between the gut microbiota and skeletal muscle in healthy individuals, but less is known about this association in populations with possible sarcopenia. More research is necessary to determine whether exercise training can alter the gut microbiota and microbial-derived chemicals linked to age-related skeletal muscle deterioration.

## Methods and design

### Ethics statement

This study was supported by the National Key Research and Development Program of China (2020YFC2002900), which was registered with the Chinese Clinical Trial Registry (ChiCTR2200064801) on Oct. 19, 2022, and approved by the Sports Science Experiment Ethics Committee of Beijing Sport University (registration number: 2020082H). Every procedure was carried out strictly in accordance with the CONSORT 2010 standards and the approved study protocol. The use of biological materials and clinical data was authorized by the institutional review board of each participating institution.

### Research design

**Figure 1:**
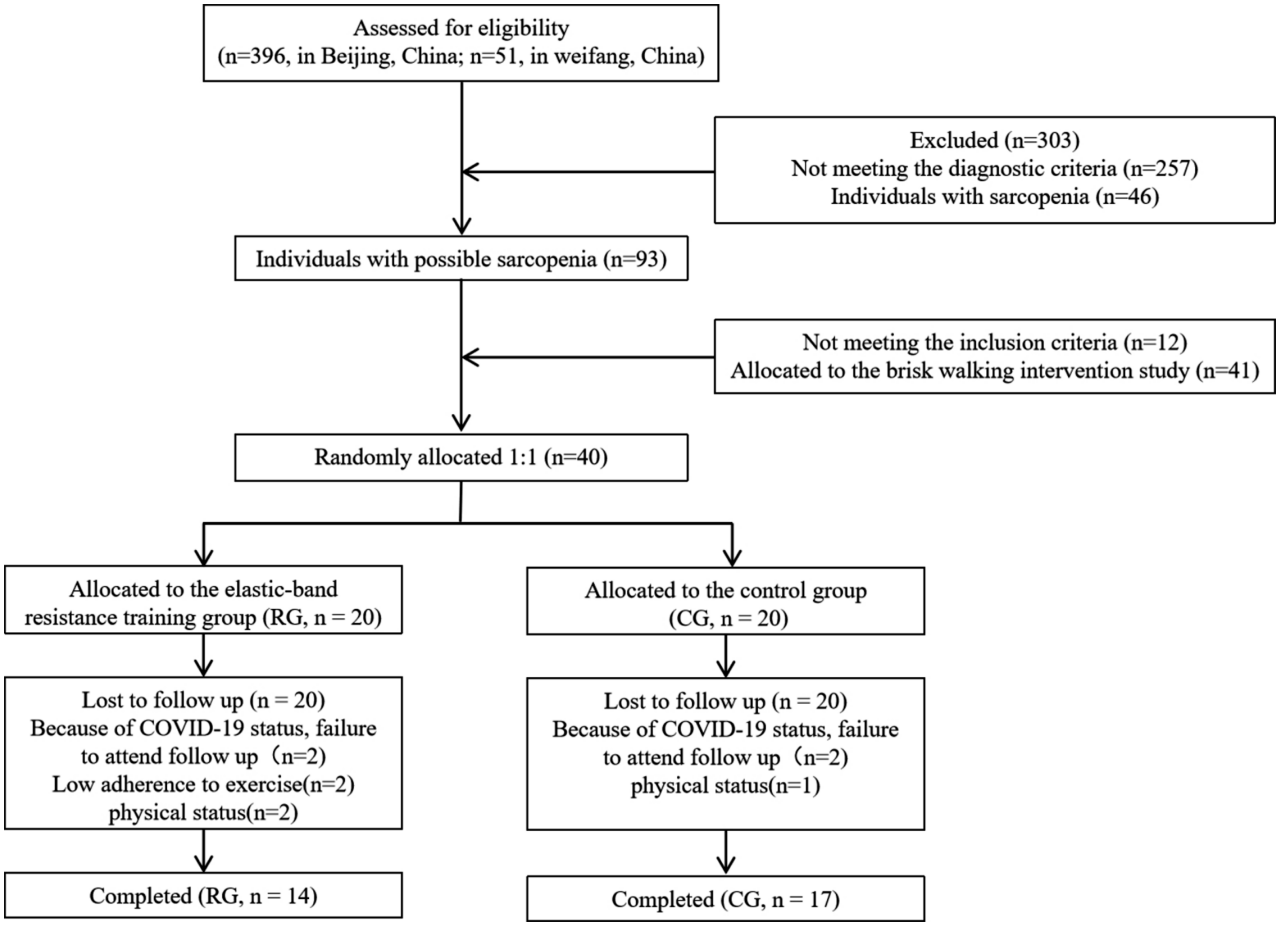
Flow chart of participants’ enrollment.

This prospective, observational intervention study was carried out at Yanda care facilities located in Beijing, China, spanning the period from 2022 to 2023. Participants had to meet the same inclusion and exclusion criteria as in our earlier study (13), and in consistent progressive resistance training within the preceding 24 weeks. Prior to formal enrollment, the prospective participant was properly informed about the goals, procedures, and possible dangers of the study and provided written informed permission (Figure 1).

To detect possible sarcopenia, the following crucial parameters were assessed in compliance with the 2019 AWGS criteria (7): Low physical performance is defined as a 6-meter walk gait speed (GS)<1.0 m/s, a 5-time chair stand test (CS-5) completion time≥12 s, or a Short Physical Performance Battery (SPPB) score ≤9. Low muscle strength is defined as a handgrip strength (HGS) <28 kg in males and <18 kg in females. Possible sarcopenia was diagnosed when an individual met either or both of the above two criteria (① and/or ②). A total of 40 elderly adults diagnosed with possible sarcopenia were enrolled in the study and randomly divided into two groups of equal size: the control group (CG, *n* = 20) and the elastic-band resistance training group (RG, *n* = 20).

### Exercise prescription and protocol

The RG participants received progressive elastic-band resistance training using THERABAND® resistance bands (Thera-Band, United States), while the CG did not participate in any structured training programs. Detailed training parameters were established as follows: ① Frequency: 3 d·wk-1. ② Intensity: Exercise loads were individualized based on the elastic resistance of the bands (indicated by band color), set at 8–12 repetition maximum (RM). THERABAND^®^ bands are color-coded in the following order: yellow, red, green, blue, black, and silver, which represent increasing elasticity and resistance levels. ③ Time: 60 min every day, divided into 40 min of core exercise training, a warm-up and cool-down. ④ Type: The training regimen used elastic bands to target all of the major muscle groups. It included stomach crunches and flexion/extension exercises for the shoulder, elbow, knee, and ankle. Following joint mobility and stretching exercises, participants engaged in upper-body exercises (seated shoulder flexion, seated shoulder extension, seated elbow flexion, seated elbow extension), lower-body exercises (seated knee extension, seated knee flexion, seated ankle flexion, seated ankle extension), and finally, abdominal crunches. Ten minutes of stretching and relaxation activities were part of the cool-down phase. ⑤ Volume: a 24-week intervention consisting of 2–3 sets of 8–12 repetitions per exercise. ⑥ Progression: Based on recurring RM testing, exercise volume was gradually increased through intensity modifications. By switching to bands of greater resistance, resistance intensity was increased (e.g., from yellow to red and then to black). A certified technician with a degree in Sports and Exercise Science trained and oversaw each participant. All participants in both groups had their attendance records kept up to date for the duration of the trial. Every participant was also told to follow their usual eating routines exactly as they were. At baseline (T0) and 24 weeks after the intervention (T1), the following outcome factors were measured: skeletal muscle mass, microbial-derived SCFAs, fecal microbiota composition, and physical function markers.

### Demographic information

A specially made questionnaire was developed to collect demographic information and assess their food intake. Sex, age, pre-existing conditions, current medical issues, and lifestyle choices were all included in the demographic information. Investigators specifically asked about the frequency of consumption of important foods, such as eggs, red meat, poultry, freshwater fish, seafood, soy products, and milk, for dietary evaluation. To aid with later laboratory tests, venous blood and fecal samples were also taken from each subject.

### Body composition and physical function measurements

Participants’ muscle mass (kg) was measured while they stood using an InBody 720 analyzer (InBody Co. Ltd., Seoul, Republic of Korea). Four electrodes were affixed to the upper and lower extremities, respectively, to estimate appendicular and trunk muscle mass after height and body weight measurements. The total of the muscle masses in the arms and legs was used to compute appendicular lean muscle mass (ALM). By dividing ALM by height squared, the appendicular skeletal muscle mass index (ASMI) was calculated (14). A series of physical performance tests, including the HGS, GS, CS-5, SPPB, 6-min walking test (6MWT), arm curl test (ACT), 30-s chair stand test (CST), 2-min step test (2MST), timed up-and-go test (TUGT), and 5-yard walking test, were used to assess functional status. Every physical function evaluation was carried out in accordance with previously defined procedures (11, 13).

Participants self-collected fecal samples, which were then chilled at home for a maximum of 24 h before being kept at −80 °C until DNA was extracted. Following the manufacturer’s instructions to the letter, 15 mL of fecal material was used for DNA extraction using the Microbiome Sampling Kit (BGI Research, Shenzhen, China). To preserve sample integrity, the processed samples were then shipped to BGI-Shenzhen on dry ice.

A Covaris ultrasonicator was used to randomly shear DNA samples into fragments. Magnetic bead-based size selection was then used to enrich for DNA fragments with an average length of 200–400 base pairs (15). A series of library construction processes, including end polishing, 3′-terminal adenylation, adapter ligation, PCR amplification, and magnetic bead-based purification, were applied to the size-selected DNA fragments (15). The final sequencing library was created by heat-denaturing double-stranded PCR amplicons and circularizing them with a splint oligonucleotide to produce single-stranded circular DNA. Following quality control (QC) verification, the qualified libraries were subjected to sequencing on the MGISEQ-2000 platform (BGI, Shenzhen, China), yielding 150-base pair (bp) paired-end reads (15). Following quality filtering, high-quality reads were assembled using the MEGAHIT assembler. Taxonomic annotation of DNA reads was performed using Kraken2, with reference to the Unified Human Gastrointestinal Genome (UHGG) database specifically curated for human stool samples. Functional annotation of the sequencing data was achieved by mapping the alignment results to the Kyoto Encyclopedia of Genes and Genomes (KEGG) database (15).

### Blood sample collection

For the quantification of plasma metabolites and serum biochemical parameters, venous blood samples were collected from the forearm of overnight-fasted participants on the same day as the physical function evaluation. Plasma and serum fractions were then isolated via centrifugation at 3,000 rpm for 30 min at 4 °C. Immediately after separation, the samples were stored at −80 °C for subsequent analyses (16).

### Plasma SCFAS quantification

For plasma SCFA quantification, 150 mL of 50% acetonitrile aqueous solution (v/v) (containing [2H9]-pentanoic acid and [2H11]-hexanoic acid as internal standards) was added to 150 mL of plasma. Following ultrasonic extraction for 10 min, the mixture was centrifuged at 12,000 rpm for 10 min. A total of 80 mL of the resulting supernatant was transferred to an injection vial. Subsequent to derivatization of both samples and standard solutions, SCFA concentrations were determined using liquid chromatography-mass spectrometry (LC–MS). The analytical system consisted of a high-performance liquid chromatography (HPLC) instrument (Nexera UHPLC LC-30A, Shimadzu, Japan) coupled with a highly sensitive mass spectrometer (AB Sciex Qtrap 5,500, United States) (17).

### Serum biochemical factors

Enzyme-linked immunosorbent assay (ELISA) was utilized to determine the serum concentrations of myostatin, insulin-like growth factor 1 (IGF-1), and tumor necrosis factor-*α* (TNF-α). Specifically, serum IGF-1 and TNF-α levels were quantified using ELISA kits procured from Shanghai, China (Human Insulin Like Growth Factor 1 ELISA Kit and Human TNF-α ELISA Kit, respectively). For myostatin measurement, an ELISA kit from a distinct manufacturer was employed (Human Myostatin (MSTN) ELISA Kit, Wuhan, China), adhering to the respective kit protocols.

### Statistical analysis

The means, standard deviations (SDs), and 95% confidence intervals (CIs) are used to express the data. The characteristics and outcomes of the participants in the RG and CG at T0 were compared using the Mann–Whitney U test, while the chi-square test was used for food frequency, alcohol consumption history, disease history, and exercise habits between groups (IBM SPSS Statistics 23.0). The interaction effect on the group’s parameters by time during the intervention periods was ascertained using a repeated-measures analysis of covariance (ANCOVA). The allocated group (RG vs. CG) was the between-groups component in the ANCOVA, whereas time was the repeated-measures factor (T0 and T1) (IBM SPSS Statistics 23.0). The Wilcoxon rank sum test was used to test the metagenome’s *α*-diversity, and the Chao1 index, ACE index, Shannon index, and Simpson index were used to show the results. The Bray-Curtis distance was used to test for *β*-diversity, which is shown as the principal coordinate analysis (PCoA) results.^1^ The effect size linear discriminant analysis (LEfSe), Wilcoxon rank sum test, and nonparametric factorial Kruskal-Wallis test were used for visualization.^2^ The relationships among fecal microbiota, skeletal muscle mass, microbial-derived SCFAs, and physical function indices were evaluated using Spearman’s correlation analysis (IBM SPSS Statistics 23.0). Statistical significance was set at a two-tailed *p* value <0.05.

## Results

### Characteristics of the participants

A total of 40 participants were enrolled and allocated to either the RG or the CG at T0. Because of their physical status or COVID-19 status, six subjects in the RG dropped out, and three subjects in the CG did not participate in the T1 post test. Seventeen subjects in the CG and fourteen subjects in the RG were included in the analysis of the results. The proportion of male participants was relatively low across all groups, with no statistically significant differences observed between groups. The participants had approximately 3 categories of comorbidities and used 4 types of regular medications, reflecting a typical elderly population with multiple chronic conditions.

Table 1 lists the specific questionnaire responses for age, food frequency, alcohol consumption history, disease history, and exercise habits of participants. No statistically significant differences in baseline parameters were observed among the groups.

**Table 1:** The main details characteristics.

Basics	CG	RG	*p* value
Age (y)	84.53 ± 7.09	82.57 ± 4.09	0.356
Sex (male/female)	6/11	5/9	0.981
Alcohol-drinking history (yes/no)	2/15	2/12	0.835
Falling (yes/no)	3/14	0/14	0.098
Exercise habit (yes/no)	16/1	10/4	0.087
Number of medications	3.24 ± 2.77	4.00 ± 2.63	0.441
Number of diseases	2.59 ± 1.33	2.86 ± 1.70	0.625
Rice (times/week)	3.94 ± 1.64	4.43 ± 1.79	0.435
Flour-based food (times/week)	3.59 ± 1.54	3.50 ± 1.45	0.872
Tubers (times/week)	4.94 ± 1.34	5.57 ± 1.02	0.159
Eggs (times/week)	3.12 ± 0.49	3.14 ± 0.53	0.892
Red meat (times/week)	4.47 ± 1.18	3.71 ± 0.91	0.059
Poultry food (times/week)	4.94 ± 0.89	4.64 ± 1.34	0.465
Freshwater fishes (times/week)	5.82 ± 1.51	6.43 ± 0.76	0.184
Seafood (times/week)	5.82 ± 1.13	6.43 ± 0.85	0.110
Soybean products (times/week)	5.24 ± 1.52	5.50 ± 1.45	0.627
Vegetables (times/week)	12.94 ± 2.05	13.64 ± 3.05	0.451
Fruits (times/week)	3.00 ± 0.35	3.14 ± 0.53	0.380
Milk (times/week)	3.06 ± 0.56	3.36 ± 0.93	0.277

The food frequency questionnaire results are listed in times per week, variables were analyzed by Mann–Whitney U tests and expressed as mean ±SD (standard deviation), significantly (*p* < 0.05)different between two groups. Alcohol-drinking history, sex, falling, disease history and exercise habit were represented by example (*n*) and analyzed by Chi-square test, significantly (*p* < 0.05)different between two groups.

### Body composition and physical function outcomes

The mean changes are presented in Table 2. Repeated-measures MANOVA demonstrated that there were no significant interaction effects on body composition parameters, including body mass index (BMI, body weight/height^2^, kg/m^2^), percentage body fat (PBF), or whole-body skeletal muscle mass represented by the ASMI. There were no significant interaction effects of group or time on HGS, CST, or SPPB. However, there was a significant interaction effect for group by time on the TUGT, 2MST, ACT, Walking 5 yards, CS-5, 6MWT and GS. Compared with those at T0, the physical function scores of the TUGT, ACT, 2MST, and GS in the RG were significantly improved at T1.

**Table 2:** Body composition and physical function outcomes.

Contents	T0	T1	Total	*F*	*P*	Partial η^2^
BMI (kg/m^2)^						
CG	22.72 ± 2.91	22.72 ± 2.71	22.72 ± 0.88			
RG	23.41 ± 4.04	23.62 ± 3.81	23.52 ± 0.91			
Total	23.05 ± 3.45	23.14 ± 3.26		0.376	0.545	0.014
*F*			0.404	0.368	0.549	0.013
*P*			0.530			
Partial η^2^			0.015			
PBF (%)						
CG	33.83 ± 6.29	33.23 ± 6.15	33.53 ± 1.85			
RG	28.28 ± 10.98	30.14 ± 7.54	29.21 ± 1.92			
Total	31.88 ± 7.21	31.74 ± 6.91		0.047	0.831	0.002
*F*			2.001	0.628	0.435	0.023
*P*			0.169			
Partial η^2^			0.069			
ASMI (kg/m^2^)						
CG	5.67 ± 0.91	5.74 ± 0.99	5.71 ± 0.27			
RG	6.65 ± 1.14	6.69 ± 1.11	6.27 ± 0.28^#^			
Total	6.14 ± 1.12	6.21 ± 1.15		2.002	0.169	0.069
*F*			6.336	0.130	0.722	0.005
*P*			0.018			
Partial η^2^			0.190			
HGS (kg)						
CG	19.91 ± 5.45	20.09 ± 4.44	20.00 ± 1.36			
RG	24.19 ± 6.44	24.74 ± 5.68	24.46 ± 1.41			
Total	21.98 ± 6.23	22.33 ± 5.52		0.524	0.475	0.019
*F*			5.025	0.125	0.726	0.005
*P*			0.033			
Partial η^2^			0.157			
TUGT (s)						
CG	9.77 ± 2.17	9.89 ± 2.22	9.83 ± 0.586	0.158	0.694	0.006
RG	9.15 ± 2.21	7.92 ± 2.27^#**^	8.54 ± 0.61	15.540	0.001	0.365
Total	9.47 ± 2.18	8.94 ± 2.42^*^		6.548	0.016	0.195
*F*	0.573	5.576	2.644	9.680	0.004	0.264
*P*	0.456	0.026	0.116			
Partial η^2^	0.021	0.171	0.089			
CS-5 (s)						
CG	10.95 ± 3.06	10.27 ± 3.28	10.61 ± 0.89			
RG	9.89 ± 3.02	8.85 ± 2.39	9.37 ± 0.92			
Total	10.44 ± 3.03	9.59 ± 2.92^**^		9.588	0.005	0.262
*F*			1.353	0.436	0.515	0.016
*P*			0.255			
Partial η^2^			0.048			
6MWT (m)						
CG	405.77 ± 99.02	384.04 ± 82.16	394.90 ± 22.51			
RG	443.50 ± 98.25	433.05 ± 110.69	438.28 ± 23.31			
Total	423.98 ± 98.76	407.69 ± 98.42^*^		5.301	0.029	0.164
*F*			1.479	0.652	0.426	0.02
*P*			0.234			
Partial η^2^			0.052			
ACT (times)						
CG	17.33 ± 7.02	16.60 ± 4.61	16.97 ± 1.14	0.313	0.580	0.010
RG	17.36 ± 2.37	18.29 ± 3.29^**^	17.82 ± 1.18	8.102	0.008	0.202
Total	15.97 ± 5.92	17.50 ± 5.33		2.168	0.151	0.063
*F*	1.441	0.761	0.057	5.330	0.028	0.143
*P*	0.239	0.390	0.813			
Partial η^2^	0.043	0.023	0.002			
CST (times)						
CG	14.57 ± 3.89	15.48 ± 4.82	15.02 ± 1.17			
RG	16.79 ± 4.54	16.86 ± 5.76	16.82 ± 1.21			
Total	15.64 ± 4.29	16.14 ± 5.24		0.524	0.475	0.019
*F*			1.193	0.382	0.542	0.014
*P*			0.284			
Partial η^2^			0.042			
2MST (times)						
CG	83.73 ± 24.81	76.20 ± 22.53	81.30 ± 5.75	3.702	0.065	0.121
RG	89.50 ± 21.70	97.57 ± 20.73^#*^	93.54 ± 5.95	3.966	0.050	0.128
Total	86.52 ± 23.13	86.52 ± 23.91		0.009	0.925	0.000
*F*	0.441	7.035	2.959	7.668	0.010	0.221
*P*	0.512	0.013	0.097			
Partial η^2^	0.016	0.207	0.099			
Walking 5 yards (s)						
CG	3.75 ± 0.79	3.48 ± 0.73	3.61 ± 0.17			
RG	3.44 ± 0.62	2.77 ± 0.85	3.11 ± 0.18^#^			
Total	3.60 ± 0.72	3.14 ± 0.85^**^		11.586	0.002	0.300
*F*			4.416	1.969	0.172	0.068
*P*			0.045			
Partial η^2^			0.141			
GS (m/s)						
CG	1.28 ± 0.32	1.29 ± 0.35	1.29 ± 0.08	0.028	0.869	0.001
RG	1.36 ± 0.25	1.80 ± 0.69^#**^	1.58 ± 0.09^#^	13.796	0.001	0.338
Total	1.32 ± 0.28	1.54 ± 0.59^**^		7.766	0.010	0.223
*F*	0.623	6.209	4.445	6.533	0.017	0.195
*P*	0.437	0.019	0.044			
Partial η^2^	0.023	0.187	0.141			
SPPB (score)						
CG	10.47 ± 2.03	10.27 ± 2.31	10.367 ± 0.46			
RG	10.64 ± 2.09	11.36 ± 1.15	11.00 ± 0.48			
Total	10.55 ± 2.03	10.79 ± 1.89		0.491	0.489	0.018
*F*			1.015	1.553	0.223	0.054
*P*			0.323			
Partial η^2^			0.036			

Data are expressed as mean ± SD (standard deviation). Variables were analyzed by repeated measure ANCOVA, significantly (*p* < 0.05)different between two groups. ^*^ Significant difference compared with T0, ^*^ *p* < 0.05; ^**^ *p* < 0.01; ^#^ Significant difference compared with the control group, ^#^ *P* < 0.05; ^##^ *p* < 0.01. BMI, body mass index; PBF, percentage body fat; ASMI, Appendicular Skeletal muscle Mass Index. HGS, handgrip strength (kg); TUGT, timed up-and-go test (s); CS-5, 5-time chair stand test (s); 6MWT, 6-min walking test (m); ACT, 30s arm curl test (times); CST, 30s-chair stand test (times); 2MST, 2-min step test (times); GS, 6-metre walk gait speed (m/s); SPPB, short physical performance battery. Gestational age of subjects represents the time at which plasma samples were collected.

### Fecal microbiota outcomes

**Figure 2:**
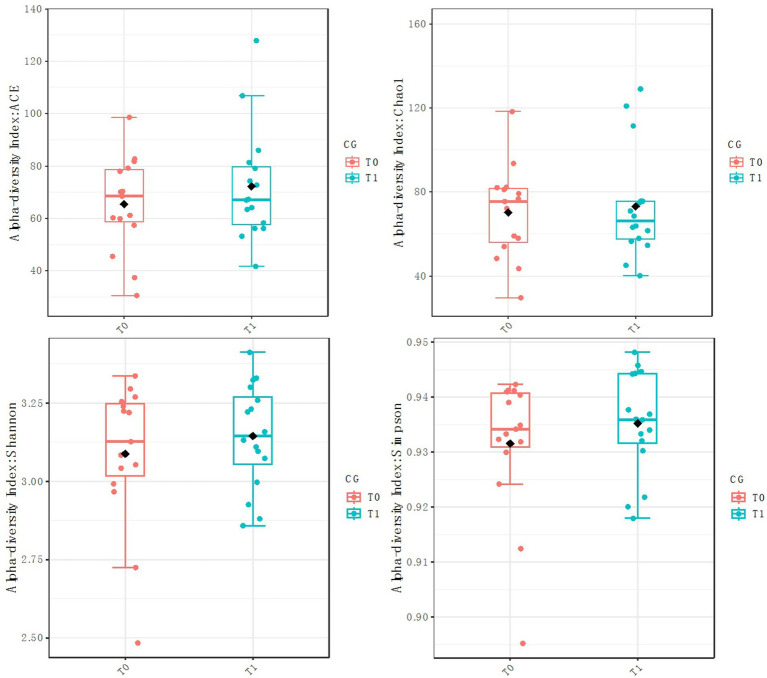
*α* diversity (CG).

**Figure 3:**
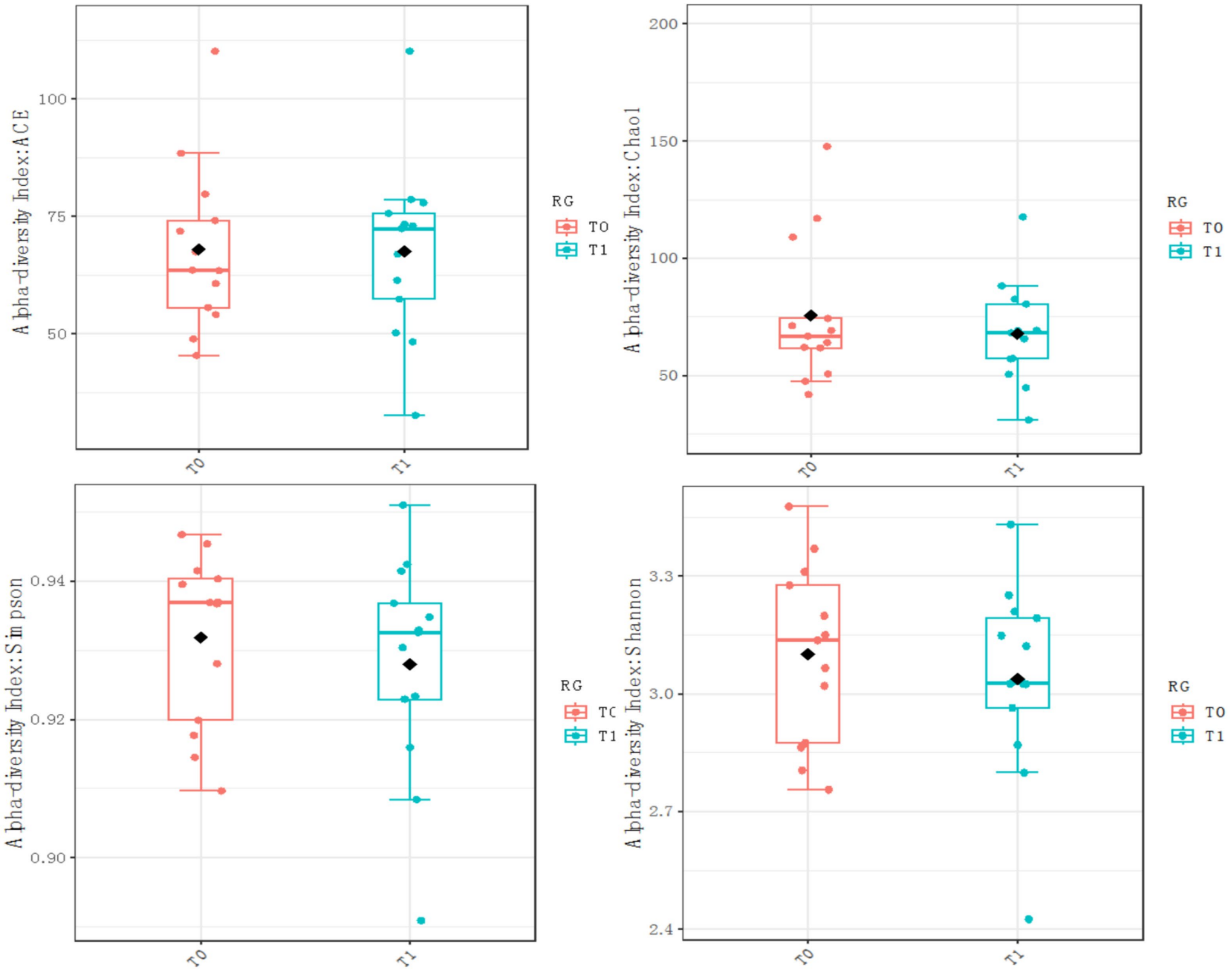
*α* diversity (RG).

The diversity of fecal microbiota was investigated via the Chao1, ACE, Shannon and Simpson indices as a measure of within-habitat diversity (*α*-diversity). The fecal microbiota diversity did not differ from T0 to T1 in the two groups. Chao1CG: *p* = 0.78, U = 127.5; ShannonCG: *p* = 0.60, U = 106; SimpsonCG: *p* = 0.45, U = 100; ACECG: *p* = 0.71, U = 110 (Figure 2). Chao1RG: *p* = 0.80, U = 90; ShannonRG: *p* = 0.61, U = 95; SimpsonRG: *p* = 0.48, U = 99; ACERG: *p* = 0.88, U = 81 (Figure 3).

**Figure 4:**
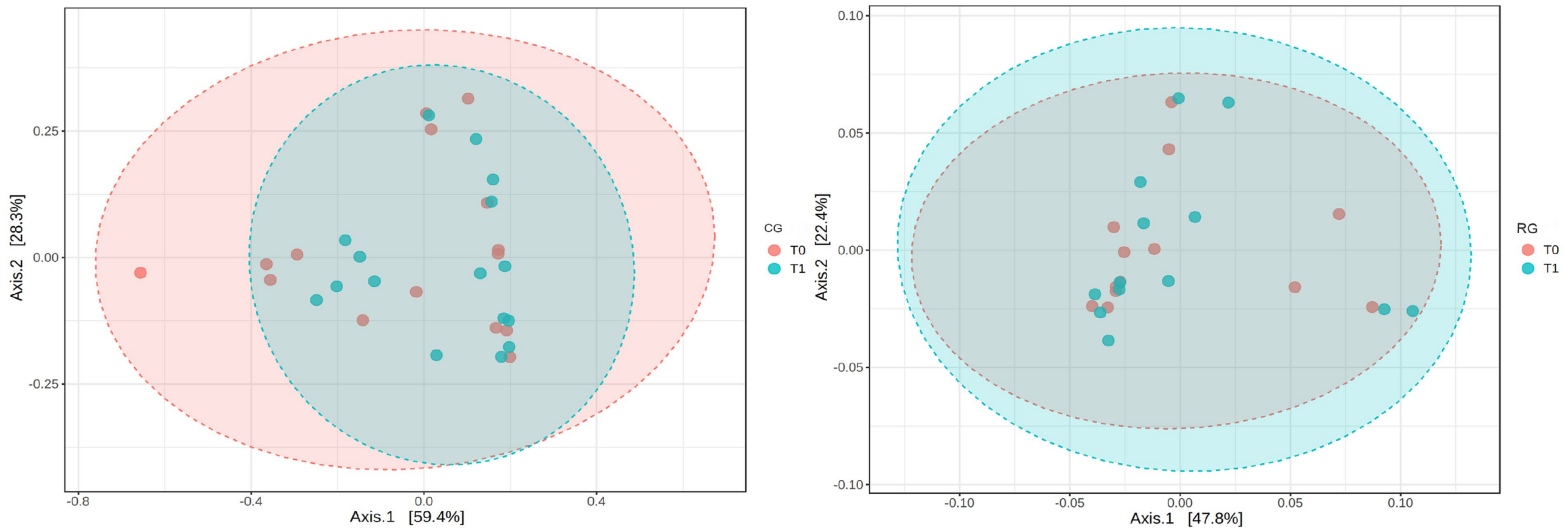
*β* diversity.

PCoA was performed to analyze the influence of exercise on the microbiota distribution at the species level (*β*-diversity). According to PCoA, there was no significant change from T0 to T1 for either group (PCoACG: *p* = 0.41, *F* = 0.96; PCoARG: *p* = 0.87, *F* = 0.31) (Figure 4).

**Figure 5:**
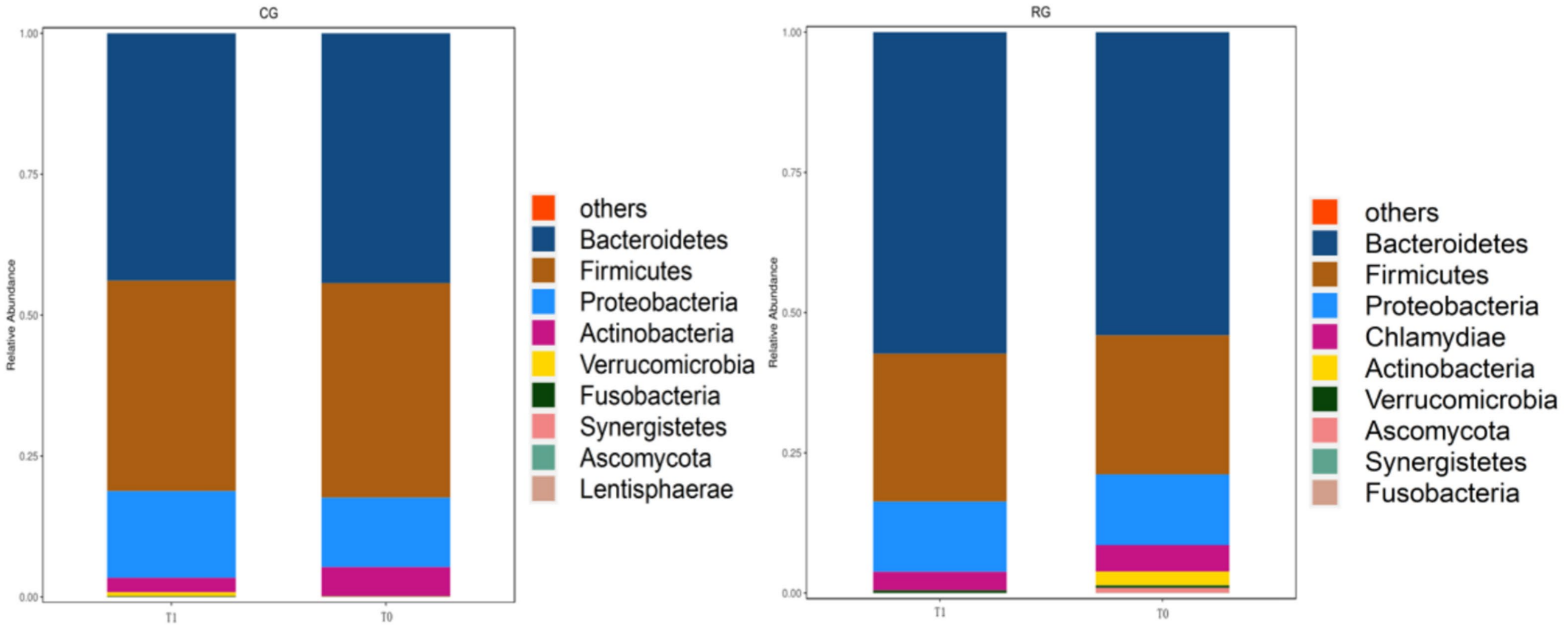
Stacked bar graph.

The three major phyla were *Bacteroidetes*, *Firmicutes* and *Proteobacteri*, and the relative abundance percentage changed from 42.45, 36.45, and 11.81% to 45.75, 38.89, and 16.03% in the CG and from 54.03, 24.21, and 12.59% to 57.32, 26.36, and 12.53% in the RG. At T1, the *Proteobacteri* of the CG presented a greater relative abundance than those observed at T0 did, suggesting that, independent of diet and exercise, age is an important modulatory factor for microbiota (Figure 5; Table 3).

**Table 3:** The relative abundance of major phyla.

Contents	T0	T1	Total	*F*	*P*	Partial η^2^
Bacteroidetes						
CG	42.45 ± 5.42	45.75 ± 3.69	44.10 ± 3.21			
RG	54.03 ± 6.01	57.32 ± 4.09	55.68 ± 3.57			
Total	48.24 ± 4.05	51.54 ± 2.75		0.436	0.514	0.016
*F*			5.814	0.000	0.999	0.000
*P*			0.023			
Partial η^2^			0.177			
Firmicutes						
CG	36.45 ± 4.56	38.89 ± 3.57	37.67 ± 2.97			
RG	24.21 ± 5.06	26.36 ± 3.96	25.58 ± 3.29			
Total	30.63 ± 3.40	32.63 ± 2.67		0.225	0.639	0.008
*F*			7.457	0.011	0.916	0.000
*P*			0.011			
Partial η^2^			0.216			
Proteobacteria						
CG	11.81 ± 1.05	16.03 ± 0.92^**^	13.92 ± 0.734	10.253	0.003	0.275
RG	12.59 ± 1.17	12.53 ± 1.02^#^	12.56 ± 0.82	0.002	0.967	0.000
Total	12.19 ± 0.78	14.28 ± 0.69^*^		4.463	0.044	0.142
*F*	0.249	6.498	1.535	4.731	0.039	0.149
*P*	0.622	0.017	0.226			
Partial η^2^	0.009	0.194	0.054			

Data are expressed as mean ±SD (standard deviation). Variables were analyzed by repeated measure ANCOVA, significantly (*p* < 0.05) different between two groups. ^*^ Significant difference compared with T0, ^*^ *P* < 0.05; ^**^ *P* < 0.01; ^#^ Significant difference compared with the control group, ^#^ *P* < 0.05; ^##^ *P* < 0.01.

**Figure 6:**
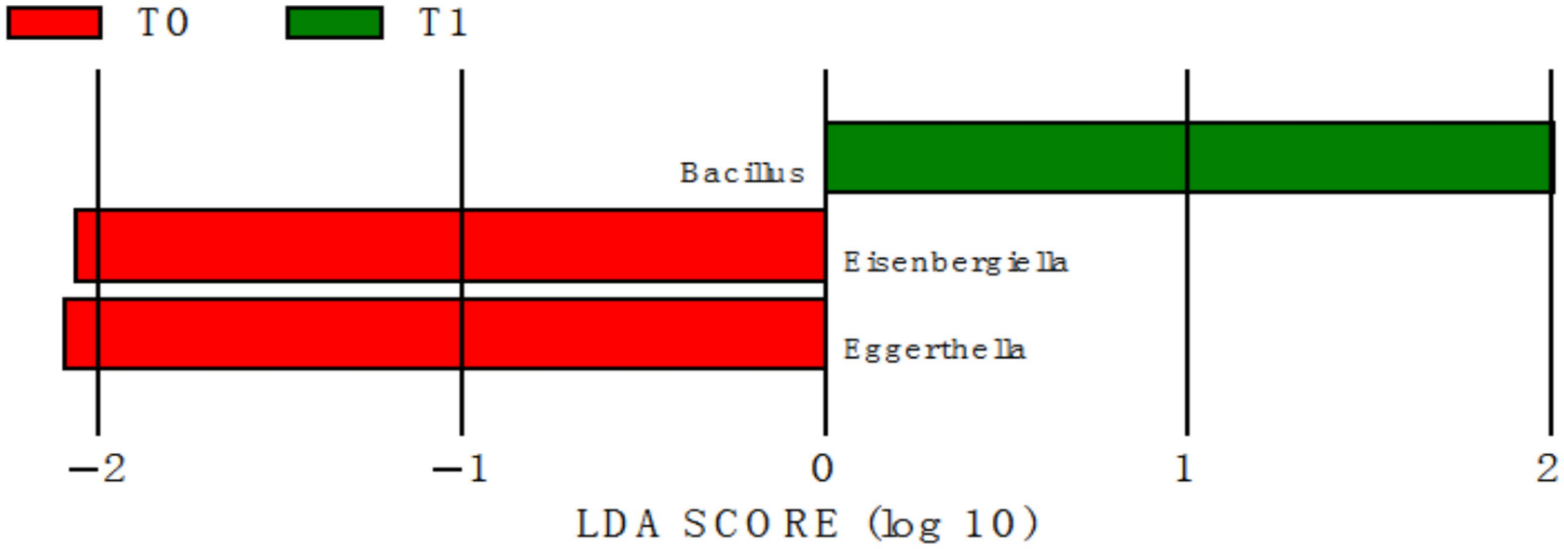
LEfSe scores.

LEfSe analysis was conducted using elastic-band resistance training intervention participation as a predictor. Accordingly, exercise modified the gut microbiome at the genus level. The genera *Eggerthella* and *Eisenbergiella* were significantly enriched at T0, and the relative abundance of *Bacillus* genera increased at T1 in the RG, but that in the CG did not change (Figure 6).

### Microbial-derived SCFA outcomes

Significant interaction effects between the groups were observed over time for acetate, propionate, and caproate, while repeated-measures MANOVA revealed no significant interaction effects for isobutyrate, butyrate, isovalerate, or valerate. At T1, the acetate and propionate contents were significantly increased in the RG, while the propionate content was significantly decreased in the CG (Table 4).

**Table 4:** Microbial-derived SCFAs outcomes.

SCFAs (ng/ml)	T0	T1	Total	*F*	*P*	Partial η^2^
Acetate						
CG	10701.21 ± 6434.92	11166.03 ± 4182.53	10933.62 ± 9399.24	0.238	0.629	0.008
RG	7205.82 ± 2679.21	11006.35 ± 3352.99^**^	9106.08 ± 1086.17	13.094	0.001	0.311
Total	8953.52 ± 921.26	11086.19 ± 691.66^**^		9.044	0.005	0.238
*F*	3.599	0.013	1.552	5.532	0.026	0.160
*P*	0.068	0.909	0.223			
Partial η^2^	0.110	0.000	0.051			
Propionate						
CG	493.65 ± 58.61	323.18 ± 44.32^**^	408.41 ± 23.46	13.705	0.001	0.321
RG	349.91 ± 270.82^*^	490.37 ± 44.11^##*^	420.14 ± 25.85	7.662	0.010	0.209
Total	421.78 ± 33.65	406.77 ± 7.98		0.192	0.665	0.007
*F*	4.561	109.697	0.113	20.591	0.000	0.415
*P*	0.041	0.000	0.739			
Partial η^2^	0.136	0.791	0.004			
Isobutyrate						
CG	111.22 ± 9.64	120.09 ± 12.23	115.66 ± 5.03			
RG	124.16 ± 38.99	126.30 ± 24.88	125.23 ± 4.18			
Total	117.69 ± 4.88	123.20 ± 3.42		0.774	0.386	0.026
*F*			2.873	0.288	0.595	0.010
*P*			0.101			
Partial η^2^			0.090			
Butyrate						
CG	273.19 ± 26.57	300.24 ± 36.39	286.72 ± 7.11			
RG	306.04 ± 92.12	316.61 ± 67.82	311.33 ± 10.61			
Total	289.62 ± 11.69	308.43 ± 9.54		1.417	0.244	0.047
*F*			2.949	0.272	0.606	0.009
*P*			0.097			
Partial η^2^			0.092			
Isovalerate						
CG	60.71 ± 11.19	76.10 ± 48.88	68.41 ± 14.89			
RG	180.17 ± 166.94	202.55 ± 60.49	191.36 ± 18.63^##^			
Total	120.44 ± 20.23	139.33 ± 9.82		0.945	0.339	0.032
*F*			23.876	0.032	0.859	0.001
*P*			0.000			
Partial η^2^			0.452			
Valerate						
CG	39.99 ± 8.39	48.16 ± 9.14	44.08 ± 3.68	1.338	0.257	0.044
RG	59.64 ± 40.58	45.15 ± 15.83	52.39 ± 4.28	3.473	0.073	0.107
Total	49.82 ± 5.03	46.65 ± 2.27		0.363	0.551	0.012
*F*	3.812	0.441	2.073	4.655	0.039	0.138
*P*	0.061	0.512	0.161			
Partial η^2^	0.116	0.015	0.067			
Caproate						
CG	512.62 ± 41.69	432.12 ± 81.39	472.37 ± 14.02			
RG	430.45 ± 94.26	407.15 ± 95.53	418.80 ± 16.13^#^			
Total	471.54 ± 12.69	419.63 ± 15.88^*^		7.660	0.010	0.209
*F*			6.047	2.325	0.138	0.074
*P*			0.020			
Partial η^2^			0.173			

Data are expressed as mean ±SD (standard deviation). Variables were analyzed by repeated measure ANCOVA, significantly (*p* < 0.05)different between two groups. ^*^ Significant difference compared with T0, ^*^ *P* < 0.05; ^**^ *P* < 0.01; ^#^ Significant difference compared with the control group, ^#^ *P* < 0.05; ^##^ *P* < 0.01.

### Plasma biochemical factors outcomes

Repeated-measures MANOVA revealed no significant interaction effects on myostatin or IGF-1. There were significant group-by-time interaction effects for TNF-*α*. The TNF-α level was significantly decreased in the RG at T1 (Table 5).

**Table 5:** Plasma biochemical factors outcomes.

Biochemical factors	T0	T1	Total	*F*	*P*	Partial η^2^
TNFα						
CG	157.52 ± 91.39	144.67 ± 87.11	151.09 ± 24.87	0.484	0.493	0.020
RG	235.01 ± 98.93^#^	163.59 ± 119.92^**^	199.30 ± 26.87	12.809	0.002	0.348
Total	196.27 ± 18.67	154.13 ± 20.35^**^		9.603	0.005	0.286
*F*	4.306	0.216	1.733	4.638	0.042	0.162
*P*	0.049	0.646	0.200			
Partial η^2^	0.152	0.009	0.067			
Myostatin						
CG	6.92 ± 3.11	7.49 ± 2.89	7.21 ± 0.75			
RG	7.10 ± 3.57	6.54 ± 2.94	6.82 ± 0.81			
Total	7.00 ± 0.65	7.02 ± 0.57		0.000	0.984	0.000
*F*			0.124	1.043	0.317	0.042
*P*			0.728			
Partial η^2^			0.005			
IGF-1						
CG	949,641 ± 29665.54	90643.68 ± 27086.56	92804.24 ± 6720.17			
RG	90538.62 ± 41585.56	97063.84 ± 28960.34	93801.23 ± 7405.26			
Total	92751.71 ± 6407.38	93853.76 ± 5042.21		0.039	0.844	0.001
*F*			0.010	0.949	0.338	0.032
*P*			0.922			
Partial η^2^			0.000			

Data are expressed as mean ±SD (standard deviation). Variables were analyzed by repeated measure ANCOVA, significantly (*p* < 0.05)different between two groups. ^*^ Significant difference compared with T0, ^*^ *P* < 0.05; ^**^ *P* < 0.01; ^#^ Significant difference compared with the control group, ^#^ *P* < 0.05; ^##^ *P* < 0.01.

### Correlation analysis outcomes

**Figure 7:**
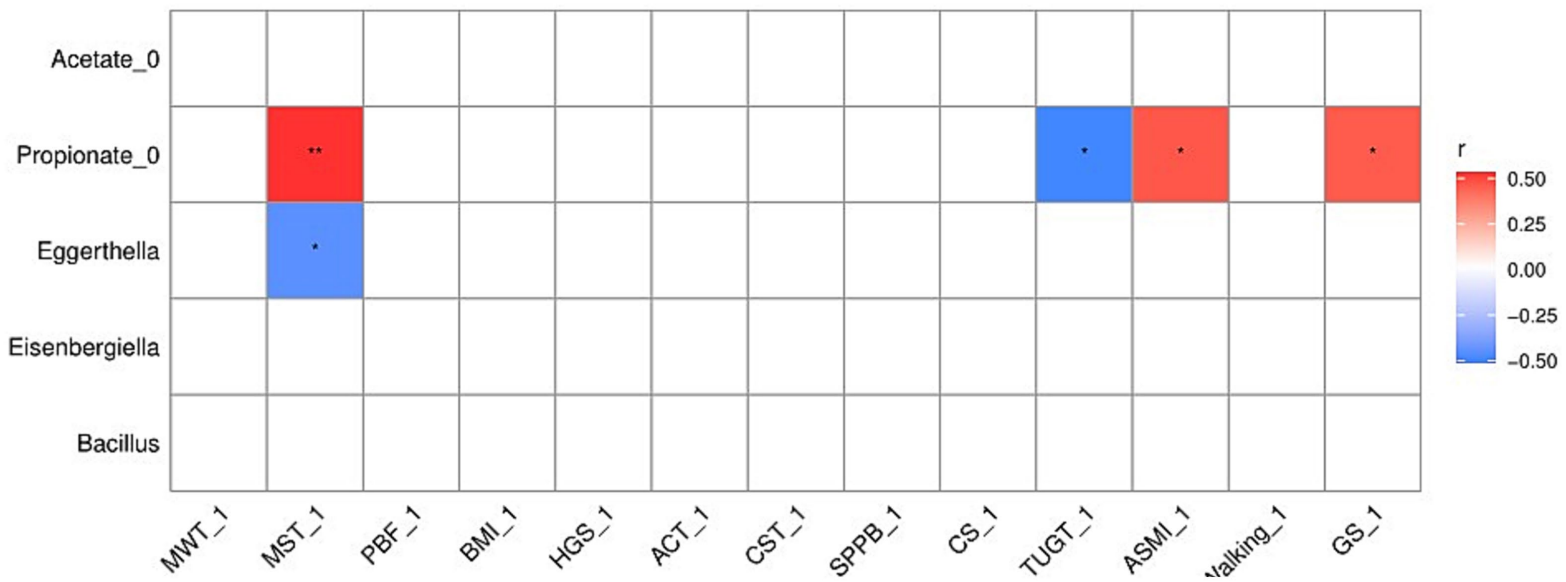
Spearman’s correlation analysis outcomes.

Spearman’s correlation coefficients were computed to examine the possible effects of fecal microbiota and microbial-derived SCFAs on skeletal muscle mass and physical function metrics. Propionate was positively associated with the 2MST (r = 0.52, *p* = 0.01), TUGT (r = 0.50, *p* = 0.01), GS (r = 0.44, *p* = 0.03), and ASMI (r = 0.45, *p* = 0.02), while *Eggerthella* was negatively associated with the 2MST (r = −0.46, *p* = 0.02), according to Spearman’s correlation analysis (Figure 7).

## Discussion

In the present study, we provide evidence that a 24-week elastic-band resistance training intervention modulates fecal microbiota and microbial-derived SCFAs in elderly individuals with possible sarcopenia, accompanied by significantly improved physical function outcomes. Additionally, we demonstrate that changes in skeletal muscle function are associated with exercise-induced alterations in fecal microbiota and microbial-derived SCFAs, highlighting the critical role of the gut microbiota in regulating physiological responses to exercise training.

### The interventional effect on aging skeletal muscle

Physical function and mobility were greatly improved by elastic-band resistance training, but body composition parameters, such as relative skeletal muscle mass, did not show any increases. Serum levels of IGF-1 and myostatin, two important regulators of muscle protein metabolism, consistently showed no discernible alterations. By preventing myoblast proliferation and differentiation, myostatin functions as a negative regulator of skeletal muscle growth and homeostasis (18). These results are consistent with other research on low- to moderate-intensity elastic-band strength training, which found that post-intervention muscle quality significantly improved but muscle mass did not (1, 19). Research indicates that in order to increase muscle mass, high training loads must be maintained for long enough periods of time (20). On the other hand, as both high- and low-intensity training produce similar benefits, changes in physical function seem to be independent of intensity (21). Our research further shows that 24-week elastic-band resistance training reduces age-related functional loss in older persons with potential sarcopenia by improving physical function as measured by TUGT, ACT, 2MST, and GS. Sarcopenia (loss of skeletal muscle), a higher likelihood of declining physical function, and decreased mobility are all linked to aging (22, 23). In this population, low muscle function and physical performance are significant predictors of unfavorable clinical outcomes. Our study’s noteworthy conclusion is that functional performance can be improved without corresponding increases in muscle mass. This is in line with earlier assessments of 6-month intervention studies that found gains in neuromuscular control abilities, muscle strength, endurance, and balance that were not related to changes in muscle mass (24). Additionally, multiple studies have demonstrated that older adults (with or without sarcopenia) exhibit a greater percentage increase in muscle strength than in skeletal muscle mass following resistance training (25, 26). These results imply that muscle mass improvements through muscle protein synthesis may not be the only factor contributing to the positive effects of elastic-band resistance exercise on mitigating muscle-related physical deficits. Changes in tissue properties, energy metabolism, neural variables, and adaptive changes in physicochemical parameters—such as fecal microbiota and microbial-derived SCFAs—that were covered in this study are some possible answers. The indirect effects of these exercise-induced adaptations warrant further investigation, as muscle quality may represent a more plastic endpoint in this elderly population. From a clinical perspective, we recommend goal-oriented exercise interventions: moderate-intensity exercise can be prioritized for improving activities of daily living and maintaining independence, particularly in elderly individuals with comorbidities (hypertension, hyperlipidemia, diabetes, osteoporosis, non-acute cardiac diseases) and functional impairments, to ensure safety and compliance.

### The interventional effect on fecal microbiota diversity

Gut microbiota diversity primarily reflects the species diversity within an individual and the structural differences between groups. In the present study, no regulatory effect on fecal microbiota *α* and *β* diversity was observed following 24-week intervention. The impact of exercise on gut microbiota diversity varies depending on specific contexts (27–31). A study involving older adults found that there was almost no difference in gut microbiota *α* diversity among individuals with different exercise frequencies; however, obese participants with high exercise frequencies exhibited higher gut microbiota α diversity, which increased with elevated BMI (32). Another study indicated that obesity is associated with reduced gut microbiota diversity (33). Our previous research did not detect significant changes in gut microbiota diversity in a potential sarcopenic population (11), suggesting that the inherent α diversity of an individual’s gut microbiota may influence its response to exercise. Currently, there is compelling evidence that gut microbiota *α* and *β* diversity are more profoundly affected by high-intensity exercise than by low-intensity exercise. Among clinical populations, exercising 4–5 times per week appears to be more beneficial than exercising only 2–3 times per week (34). In the current study, although there was a trend toward dispersion in fecal microbiota β diversity in the RG after 24 week intervention, this did not reach statistical significance. The absence of a regulatory effect of elastic-band resistance training on fecal microbiota diversity in the present study indicates that the interventional impact of exercise on the gut microbiota is not entirely dependent on alterations in α or β diversity. Because diversity metrics can be influenced by a variety of factors, such as sample size, exercise modality, interindividual heterogeneity in exercise responsiveness, and intrinsic interindividual variations in the composition of the gut microbiota, relying exclusively on them to evaluate the effects of exercise on the gut microbiota may be limited.

### The interventional effect on the species composition of fecal microbiota

The makeup of the fecal microbiota varied greatly between the RG and CG, despite disagreements over the effect of exercise on fecal microbiota diversity. Interestingly, after intervention, the RG’s relative abundance of *Proteobacteria*—a phylum that includes both commensal bacteria and several pathogens—dropped. The CG, on the other hand, showed a notable rise in *Proteobacteria* abundance over time, indicating that this negative shift was avoided by elastic-band resistance training. These results are consistent with previous research showing that exercise lowers intestinal *Proteobacteria*, a potentially pro-inflammatory taxon, in high-aerobic-capacity rats and overweight women (35). Concurrently, there was a significant decrease in serum TNF-*α* levels in the RG, which could be mechanistically related to the lower abundance of *Proteobacteria*.

Exercise can change the relative abundance of particular bacterial phyla, families, or species in addition to microbial richness and diversity (36, 37). The taxa *Eisenbergiella* and *Eggerthella* were enriched in the RG at baseline, but Bacillus took over after the intervention. After 24 weeks, there were no discernible changes in the CG. *Eisenbergiella* enrichment in those who may have sarcopenia supports earlier research in humans: *Eisenbergiella* is a particular, sensitive biomarker for differentiating sarcopenia from healthy condition, and 16S rRNA gene sequencing reveals that it is markedly increased in sarcopenic individuals (38). *Eisenbergiella* also exhibits pro-inflammatory properties and is associated with chronic disease development (39), and its abundance is increased in individuals with high saturated fatty acid (SFA) intake (40). *Eggerthella*, previously linked to biological aging and higher abundance in elderly populations (41, 42), was also significantly reduced following elastic-band resistance training. This genus is a gut microbiota marker for frailty (43) and is more abundant in cirrhotic patients with sarcopenia than in non-sarcopenic cirrhotic patients (44). *Eggerthella lenta*, a pathogenic species within this genus, is associated with gastrointestinal disorders (45) and harbors a cardiac glycoside reductase operon that inactivates digoxin by converting it to lactone dihydrodigoxin (46). Notably, we found a negative correlation between *Eggerthella* abundance and 2MST performance improvements, suggesting that reduced *Eggerthella* levels are associated with enhanced physical function.

On the other hand, after the intervention, *Bacillus* abundance was considerably higher in the RG. Studies show that supplementing with *Bacillus coagulans FCYS01* maintains the expression of tight junction and mucin proteins, encourages intestinal barrier repair, modifies cytokine levels, enriches bacteria that produce SCFA (such as *Akkermansia* and *Ruminococcus*), and increases SCFA synthesis (47). *Prebiotics* and *Bacillus coagulans GBI-30, 6,086* consumption also boost the generation of organic acid and beneficial bacterial populations (48). *Bacillus subtilis* or *Bacillus licheniformis* food supplements boost growth performance, immunological function, and antioxidant capacity in chickens while also increasing SCFA synthesis and altering the cecal microbiota (49). All of these results point to the potential health benefits of exercise-induced increases in *Bacillus* and decreases in *Eisenbergiella* and *Eggerthella*.

### The interventional effect on the SCFAs

Undigested substrates are transformed into bioactive metabolites by the gut microbiota, which then enter the bloodstream and alter human physiology (50). SCFAs are important molecules that are created when bacteria ferment indigestible proteins and carbohydrates (51). Acetate, propionate, and butyrate are the three main SCFAs found in the rumen and large intestine (52). Systemic energy balance is regulated by these volatile fatty acids (less than six carbons) (53). Elastic-band resistance exercise not only changed the composition of the fecal microbiota but also raised the amounts of propionate and acetate in plasma. Increased intestinal content mixing and bacterial fermentation of dietary fiber; increased anaerobic fermentation due to changes in colonic pH or oxygen tension; decreased intestinal SCFA utilization and absorption; increased endogenous SCFA production (e.g., from lactate) (54); and improved gut microbiota SCFA-producing capacity are some of the mechanisms that may underlie exercise-induced increases in plasma SCFAs. We hypothesize that exercise-induced improvements in SCFA-producing activities lead to enhanced plasma SCFAs, which may be partially mediated by *Bacillus*, since *Bacillus* enrichment is linked to increased butyrate-producing bacteria (49). Moreover, these functional changes in the machinery involved in SCFA biosynthesis may enhance the gut reactions to environmental stimuli (such as a higher capacity for fermenting dietary fiber), which would ultimately boost the production of SCFA (55).

SCFAs are widely acknowledged as important mediators of the gut-muscle axis (56). Exercise-induced increases in plasma propionate strongly correlated with improvements in skeletal muscle quality (including lean mass). There is growing evidence that SCFAs have positive effects on skeletal muscle: supplementing with SCFAs causes an oxidative skeletal muscle phenotype in rodents fed a high-fat diet (57, 58). It has been demonstrated to lessen age-related skeletal muscle atrophy in mice on a non-obesogenic diet (59). These bacterial metabolites may be essential metabolic substrates for skeletal muscle during prolonged contraction, according to mounting data. Scheiman et al. showed that rectal propionate infusion enhances exercise capacity to a comparable degree as *Veillonella atypica* inoculation (60), supporting propionate’s ergogenic potential. In contrast to saline infusion, continuous subcutaneous acetate infusion restores endurance in antibiotic-treated C57BL/6 J mice (a model of gut microbiota depletion) (61), and 4-week chronic SCFA administration returns muscle strength in germ-free C57BL/6 J mice to levels similar to conventionally raised mice (10). Potential mechanisms include enhanced insulin sensitivity (62), decreased inflammation (63), controlled satiety (64), and SCFA-mediated regulation of skeletal muscle lipid, carbohydrate, and protein metabolism (shown *in vitro* and *in vivo*) (65). The aforementioned findings support the maintenance of an oxidative phenotype (58), improved function and exercise capacity (66), and preservation of muscle mass (67). Important molecular processes could include inhibition of histone deacetylases (HDACs) and activation of AMP-activated protein kinase (AMPK), peroxisome proliferator-activated receptor *δ* (PPAR-δ), and peroxisome proliferator-activated receptor gamma coactivator 1-*α* (PGC1α) (62). We hypothesize that exercise-induced elevation of gut microbiota SCFA-producing ability constitutes a positive adaptation that contributes to energy replenishment and improvements in physical function, integrating our findings with earlier cross-sectional research (28, 68).

In conclusion, elastic-band resistance training mitigated age-related decline in older persons with possible sarcopenia by improving physical function (TUGT/ACT/2MST/GS) without increasing muscle mass or changing serum IGF-1/myostatin levels. The intervention increased beneficial *Bacillus* and decreased pro-inflammatory taxa (*Eggerthella*, *Eisenbergiella*), but it had no effect on the α/*β* diversity of the fecal microbiota. Additionally, it raised plasma acetate and propionate levels, which were linked to improved muscle quality. By mediating gut-muscle axis crosstalk through AMPK/PPAR-*δ*/PGC1α activation and HDAC inhibition, exercise-induced *Bacillus* enrichment may increase SCFA synthesis, supporting its role in energy replenishment and function improvement. However, this study has a number of limitations: (1) dietary assessments may be subject to recall bias, even though there were no significant differences in dietary patterns between groups; (2) despite active recruitment efforts, the number of male participants remained small, necessitating cautious interpretation of results regarding sex differences; and (3) the study design only included participants who were physically capable of performing resistance training, limiting the generalizability of findings to all elderly individuals with possible sarcopenia. To overcome these constraints, future research should increase sample sizes, impose stringent food restrictions, and include more male subjects.

## Data Availability

The original contributions presented in the study are included in the article and its Supplementary material. Further inquiries can be directed to the corresponding author.

## Footnotes

1
https://www.microbiomeanalyst

2
https://www.cloudtutu.com
