# Supplementary material for: The effect of elastic-band resistance training on fecal microbiota and derived metabolites of aged individuals with possible sarcopenia
Source: Front Med (Lausanne). 2026 Mar 26;13:1762454. doi: 10.3389/fmed.2026.1762454 (PMC13062230; doi:10.3389/fmed.2026.1762454)
Supplement: Supplementary file 1 [file Data_Sheet_1.PDF]

Explanation of Discrepancies in Subject Enrollment Between the Registered Clinical Trial Protocol and the Manuscript

Title: Elastic-band resistance training alters fecal microbiota and derived metabolites in aged individuals with possible sarcopenia

Clinical Trial Registration Platform and Number: This Manuscript was supported by the National Key Research and Development Program of China (2020YFC2002900), which was registered with the Chinese Clinical Trial Registry (ChiCTR2200064801) on Oct. 19, 2022. and approved by the Sports Science Experiment Ethics Committee of Beijing Sport University (registration number: 2020082H).

A sample size of 200 individuals for the control group and an additional 200 for the intervention group was provided in the trial registration material. Nevertheless, the statistical analysis of this publication finally included forty individuals in total. The EQUATOR Network's concept of extenuating circumstances is met by the particular reasons for this disparity in subject registration and the related handling procedures. The following details are included in this explanation, which was created strictly in compliance with the CONSERVE-CONSORT guidelines:

1.The primary goal of the study endeavor that supports this publication was to enlist 400 individuals. Elastic-band resistance training and brisk walking exercises were two of the project's intervention modalities; the latter was used for 12 and 24 weeks. The results of the 24-week elastic-band resistance training intervention are the only data included in this publication. Following the study's start, 396 volunteers were found through hospital referrals and community-based screening. Below is a summary of the comprehensive enrollment and screening results:

(1) Muscle mass measurement, handgrip strength testing, and physical function assessment were used to determine eligibility. The diagnostic criteria carefully adhered to the standards developed by the Asian Working Group for Sarcopenia (AWGS) in 2019. Of the 396 recruited people, 46 were diagnosed with sarcopenia, 93 were recognized as having possible sarcopenia, and 257 were rejected for not meeting the diagnostic criteria for either sarcopenia or possible sarcopenia.

(2) An additional 52 participants were eliminated for reasons that could skew the results of the study or make it more difficult for them to follow the intervention procedure. Comorbid severe gastrointestinal disorders, concurrent use of probiotics or antibiotics that could affect fecal microbiota analysis, malignant tumors, or cognitive impairment that would make it impossible to finish the training and follow-up procedures were among the specific exclusion criteria. Of the 52 persons that were excluded, five had sarcopenia, twelve had probable sarcopenia, and thirty-five were neither sarcopenic nor possibly sarcopenic.

2.Following screening, 344 participants — 222 non-sarcopenic/non-possible sarcopenic persons, 41 patients with sarcopenia, and 81 individuals with probable sarcopenia — were successfully enrolled. Following that, these participants were divided into two sub-studies: 40 individuals with potential sarcopenia were assigned to the elastic-band resistance training group (the subject of this manuscript), while 82 participants (41 with possible sarcopenia and 41 with sarcopenia) were assigned to the brisk walking intervention group. In order to describe the gut microbiota and metabolite profiles linked to varying degrees of skeletal muscle aging, baseline comparative analyses were also carried out between the 222 non-sarcopenic/non-possible sarcopenic participants and the 122 sarcopenic/possible sarcopenic participants (81 with possible sarcopenia and 41 with sarcopenia).

3.Nine of the forty individuals in the elastic-band resistance training group were not included in the final statistical analysis because of insufficient data or noncompliance: The COVID-19 pandemic caused four participants to miss follow-up evaluations; two participants were eliminated for not following the recommended training protocol (training compliance < 75%), which prevented a reliable assessment of the intervention effect; and three participants withdrew from post-intervention testing because they reported experiencing physical discomfort. In the end, 31 participants finished the whole elastic-band intervention course and all necessary evaluations; their information was incorporated into the final analysis.
